# Diurnal Asymmetric Loading Modulates Cell Phenotype in Intervertebral Disc

**DOI:** 10.1002/jsp2.70068

**Published:** 2025-05-07

**Authors:** Ying Zhang, Jianbiao Xu, Zhiyu Zhou, R. Geoff Richards, Mauro Alini, Sibylle Grad, Zhen Li

**Affiliations:** ^1^ AO Research Institute Davos Davos Switzerland; ^2^ Department of Orthopedics, Spine Center Changzheng Hospital, Naval Medical University Shanghai China; ^3^ Department of Orthopaedics First Affiliated Hospital of Tsinghua University Beijing China; ^4^ School of Clinical Medicine Tsinghua University Beijing China; ^5^ Department of Orthopaedic Surgery The Seventh Affiliated Hospital, Sun Yat‐Sen University Shenzhen China

**Keywords:** asymmetric loading, disc degeneration, intervertebral disc, organ culture model, scoliosis

## Abstract

**Purpose:**

This study tested the hypothesis that asymmetric dynamic loading alone or in combination with static loading influences the morphological and biological characteristics of the intervertebral disc (IVD) cells in an ex vivo model.

**Methods:**

Bovine caudal IVDs were assigned to four groups: (1) Parallel dynamic load (1 h) + free swelling (23 h); (2) Parallel dynamic load (1 h) + static load (23 h); (3) Wedge dynamic load (1 h) + free swelling (23 h); (4) Wedge dynamic load (1 h) + static load (23 h). IVD structure was assessed with measurements of height loss and histological staining. IVD tissue and cellular responses were also measured.

**Results:**

Diurnal dynamic loading and free swelling recovery could maintain cell viability and the gene expression of organ‐cultured discs at their physiological level. Diurnal dynamic loading followed by static loading resulted in a degenerative condition, as indicated by lower cell viability, lower anabolic, and higher catabolic gene expression. Under the dynamic load + free swelling load regime, wedge loading upregulated the ACAN gene expression level in the concave and convex sides of the annulus fibrosus (AF) compared with day 0 healthy control. Under the dynamic load + static loading regime, the MMP1 gene expression showed a trend of increase in the concave and convex sides of the wedge group; the MMP13 gene expression showed a trend of increase in the concave side of the wedge group. The nucleus pulposus (NP) tissue in the wedge group showed a trend of protrusion toward the convex side.

**Conclusion:**

Dynamic loading followed by continuous static loading negatively modulates the phenotype of IVD cells in this organ culture model. Comparable to the free swelling treatment after dynamic loading, physical treatment to reduce the stress on the IVD, even temporarily, may help to prevent the acceleration of deformity and degeneration. These results indicate that asymmetric loading followed by static loading may be used to mimic pathological changes of the IVD in spinal deformity.

## Graphical abstract

Dynamic loading followed by continuous static loading negatively modulates the phenotype of IVD cells in an organ culture model. Comparable to the free swelling treatment after dynamic loading, physical treatment to reduce the stress on the IVD, even temporarily, may help to prevent the acceleration of deformity and degeneration. The results of this study indicate that asymmetric loading followed by static loading may be used to mimic pathological changes of the IVD in spinal deformity.

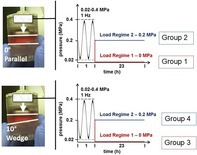

## 1 Introduction

Mechanical loading conditions are known to regulate the biological and biomechanical properties of intervertebral discs (IVD) [1, 2]. Physiological mechanical stimulation is crucial for keeping the IVD in a healthy condition [3, 4]. The main influential parameters that define the mechanical stimulus include the direction, frequency, and amplitude of the stress, and the loading duration.

Previous research demonstrated that human IVD cells, including both nucleus pulposus (NP) and annulus fibrosus (AF) cells, respond to different kinds of mechanical stress. Cyclic stretching at a frequency of 6 cycles/min could activate the RhoA/MRTF‐A signaling pathway, promote extracellular matrix degeneration, and induce inflammation in NP cells from patients with IVD degeneration [5, 6]. Compressive stress at 0.5 MPa played an anti‐angiogenic role by inhibiting endothelial cell migration in a 2D culture model [7]. These studies demonstrate that mechanical loading has a significant influence on the biological processes of human IVD. However, the method of 2D cultured cells in these studies can hardly simulate the real mechanical situation of the IVD, as it lacks the 3D environment and the combination of NP, AF, and endplates [8].

Ex vivo cultured whole IVD, with mechanical loading generated by a bioreactor, is a suitable model to investigate the impact of physiological and pathological stress on IVD, thereby compensating for the shortcomings of the cell culture model [9, 10]. Previous studies on dynamic axial compressive loading suggested that a magnitude of 0.2–0.8 MPa at a frequency of 0.1–1 Hz for a duration of up to 8 h/day lies within the physiological range, where mRNA expression levels of the IVD cells are maintained with no anabolic or catabolic shift [1].

While axial compression alone is simple and reproducible for ex vivo organ culture purposes, complex loading with the combination of multiple loading modes mimics the real spine motion situation in vivo more closely. In spinal segments ex vivo, a large range of motion at flexion/extension has been shown to increase matrix metalloproteinase (MMP)‐mediated aggrecan fragmentation in AF tissue [11]. Studies investigating the effects of compression, bending, and twisting on ovine lumbar spines have shown that complex posture significantly reduces the load required to cause disc failure [12, 13].

Yet these studies with complexed loading mainly focus on the mechanical and structural changes of IVD over time. The effect of unparallel loading, representing spinal deformity, on the biological changes of IVD has scarcely been studied. Iatridis et al. [14] showed that constant static wedge compression had direct deleterious effects on IVD tissue and cells. Intermittent asymmetric dynamic and static loading may represent a more physiological regime and lead to different outcomes. The aim of this project was to test the hypothesis that asymmetric dynamic and static loading influence the morphological and biological characteristics of the IVD cells. The study was performed with organ cultured bovine caudal IVDs using a self‐designed asymmetrical loading device.

## 2 Materials and Methods

### 2.1 Dissection of Bovine Caudal IVDs

Bovine caudal IVDs were dissected from tails of 6–10 months old animals supplied by the local abattoirs as described previously [15, 16, 17]. IVDs including the cartilaginous endplates, were harvested with a band saw (model 30/833, Exakt Apparatebau, Norderstedt, Germany) and cleaned with Ringer solution using a Pulsavac jet‐lavage system (Zimmer, IN, USA). The IVDs were then disinfected with phosphate‐buffered saline (PBS) containing 1000 U/mL penicillin and 1000 μg/mL streptomycin (all products from Gibco, Basel, Switzerland) for 15 min. The cleaned IVDs were transferred to a 6‐well plate and cultured in an incubator at the condition of 37°C, 85% humidity, and 5% CO_2_. The culture medium was composed of Dulbecco's modified Eagle medium (DMEM) containing 4.5 g/L glucose and supplied with 2% fetal calf serum, 100 units/mL penicillin, 100 μg/mL streptomycin (all products from Gibco, Basel, Switzerland), 1% ITS+ Premix (Discovery Labware Inc., Bedford, USA), 50 μg/mL ascorbate‐2‐phosphate (Sigma‐Aldrich, St. Louis, USA) and 0.1% Primocin (Invitrogen, San Diego, CA, USA). IVDs (disc height 9.9 ± 1.4 mm, disc diameter 16.4 ± 1.8 mm) were randomly assigned among experimental groups to obtain similar average disc size and distribution of disc levels for each group.

### 2.2 Loading Conditions

**FIGURE 1:**
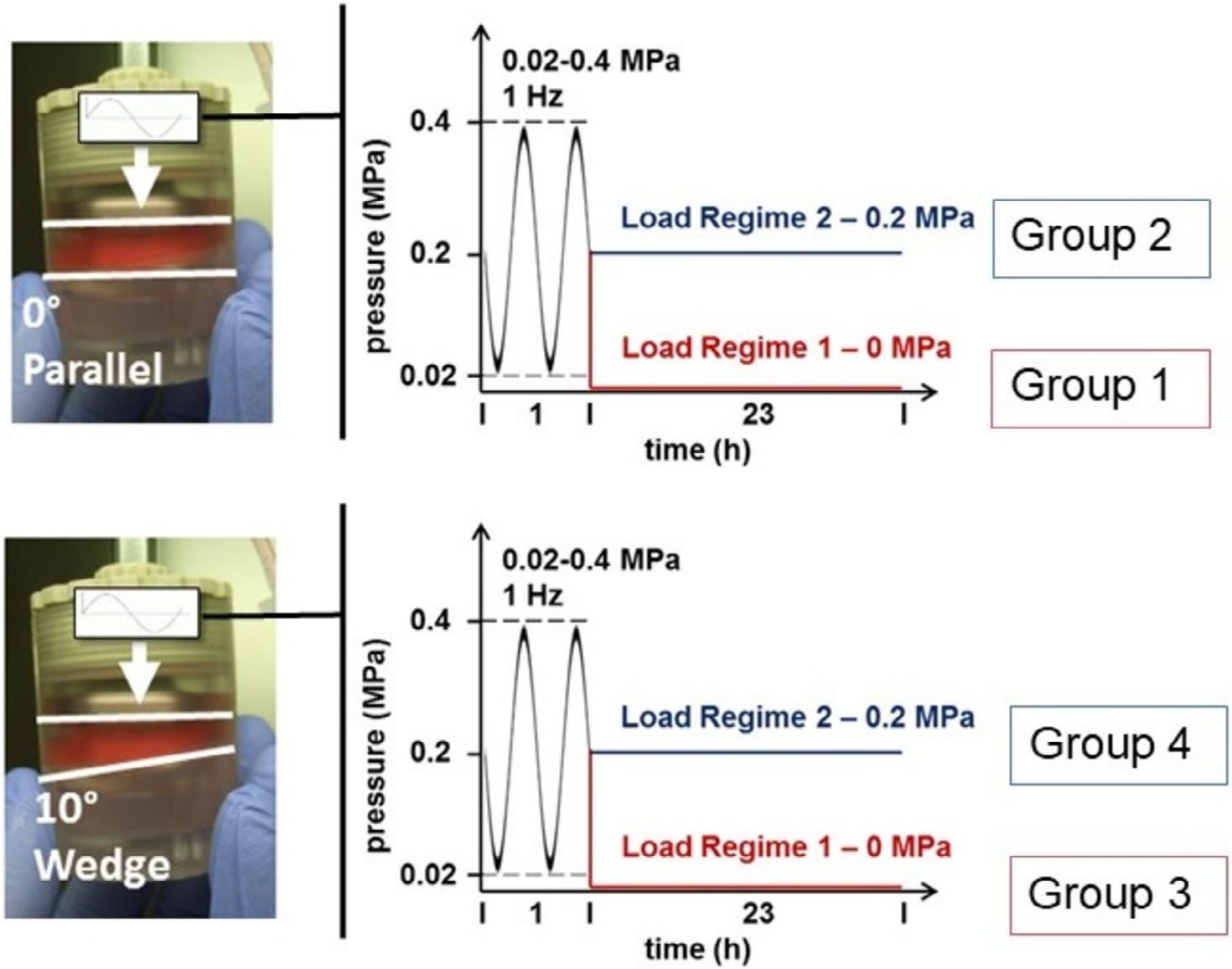
Left top: IVD within a custom‐made chamber for symmetric parallel dynamic loading. Left bottom: IVD within a custom‐made chamber for asymmetric dynamic loading by placing a 10° wedge inlet at the bottom. Right: Scheme of 4 groups with 2 different load regimes applied on IVDs.

The IVDs were assigned to 4 groups (Figure 1): (1) Parallel, dynamic load + free swelling (load regime 1); (2) Parallel, dynamic load + static load (load regime 2); (3) Wedge, dynamic load + free swelling (load regime 1); (4) Wedge, dynamic load + static load (load regime 2). For symmetric dynamic loading groups (parallel, Figure 1 left top), the IVDs were placed in custom‐designed chambers [18], and compressed by parallel metal plates in a Bose 3220 mechanical loading device (Bose, MN, USA). For asymmetric dynamic loading groups (wedge, Figure 1 left bottom), a 10° wedge inlet was placed underneath the IVDs. The IVDs in the wedge groups were marked with a skin marker at the middle upper position between the convex and the concave sides. During the culture period, no sliding or rotation of IVDs was observed, thus allowing a clear identification of the convex and concave sides. The loading protocols of the two load regimes applied were as following (Figure 1 right): Load Regime 1—1 h of sinusoidal dynamic loading at 0.02–0.4 MPa, 1 Hz, followed by 23 h free swelling culture, for 7 days; load regime 2—1 h of sinusoidal dynamic loading at 0.02–0.4 MPa, 1 Hz, followed by 23 h static loading at 0.2 MPa, for 7 days. In between the dynamic loading cycles, the IVDs were cultured in an incubator at 37°C, 85% humidity, and 5% CO_2_. Medium was changed twice a day before and after dynamic loading (5 mL/IVD).

### 2.3 Measurement of Disc Height Changes

The disc height (including cartilaginous endplates) was measured with a caliper for each IVD at different time points: on day 0 immediately after dissection, before and after dynamic load on days 1, 3, and 7. For IVDs in Parallel groups, each IVD was measured at 4 positions, and the average value was taken for the disc height. For IVDs in Wedge groups, the disc height at the convex side, concave side, and the middle position between the convex and concave sides was measured.

### 2.4 Gene Expression Analysis

After 7 days of culture with loading, the cartilaginous endplates of the IVDs were removed, and the AF and NP tissues were collected for gene expression analysis using scalpel blades and biopsy punches (~100 mg tissue/sample). For IVDs in Parallel groups, AF tissue was collected at a random position. For IVDs in Wedge groups, AF tissue was collected at the convex and concave sides. The collected tissues were digested with 2 mg/mL pronase for 1 h at 37°C [19]. Tissue was then flash frozen, pulverized in liquid nitrogen, and homogenized using a TissueLyser (Qiagen, Venlo, Netherlands). Total RNA was extracted with TRI Reagent (Molecular Research Center) and reverse transcription was performed with SuperScript VILO cDNA Synthesis Kit (Life Technologies, Carlsbad, CA). Quantitative real‐time PCR was performed using the Step‐One‐Plus instrument (Life Technologies), with 40 cycles of 15 s at 95°C for denaturation and 1 min at 60°C for annealing. The sequences of custom‐designed bovine primers and TaqMan probes are shown in Table 1. For amplification of RPLP0, a gene expression assay from Applied Biosystems (Bt03218086_m1, Life Technologies) was used. The comparative Ct method was performed for the relative quantification of target mRNA with RPLP0 as the endogenous control.

**TABLE 1:** Oligonucleotide primers and probes (bovine) used for real‐time PCR.

Gene	Gene ID	Primer/probe type	Sequence
COL1	282188	Primer fw (5′–3′)	TGC AGT AAC TTC GTG CCT AGC A
		Primer rev (5′–3′)	CGC GTG GTC CTC TAT CTC CA
		Probe (5′FAM/3′TAMRA)	CAT GCC AAT CCT TAC AAG AGG CAA CTG C
COL2	407142	Primer fw (5′–3′)	AAG AAA CAC ATC TGG TTT GGA GAA A
		Primer rev (5′–3′)	TGG GAG CCA GGT TGT CAT C
		Probe (5′FAM/3′TAMRA)	CAA CGG TGG CTT CCA CTT CAG CTA TGG
ACAN	280985	Primer fw (5′–3′)	CCA ACG AAA CCT ATG ACG TGT ACT
		Primer rev (5′–3′)	GCA CTC GTT GGC TGC CTC
		Probe (5′FAM/3′TAMRA)	ATG TTG CAT AGA AGA CCT CGC CCT CCA T
MMP1	281308	Primer fw (5′–3′)	TTC AGC TTT CTC AGG ACG ACA TT
		Primer rev (5′–3′)	CGA CTG GCT GAG TGG GAT TT
		Probe (5′FAM/3′TAMRA)	TCC AGG CCA TCT ACG GAC CTT CCC
MMP13	281914	Primer fw (5′–3′)	CCA TCT ACA CCT ACA CTG GCA AAA
		Primer rev (5′–3′)	GTC TGG CGT TTT GGG ATG TT
		Probe (5′FAM/3′TAMRA)	TCT CTC TAT GGT CCA GGA GAT GAA GAC CCC
IL1	281251	Primer fw (5′–3′)	TTA CTA CAG TGA CGA GAA TGA GCT GTT
		Primer rev (5′–3′)	GGT CCA GGT GTT GGA TGC A
		Probe (5′FAM/3′TAMRA)	CTC TTC ATC TGT TTA GGG TCA TCA GCC TCA A
IL6	280826	Primer fw (5′–3′)	TTC CAA AAA TGG AGG AAA AGG A
		Primer rev (5′–3′)	TCC AGA AGA CCA GCA GTG GTT
		Probe (5′FAM/3′TAMRA)	CTT CCA ATC TGG GTT CAA TCA GGC GATT

**Abbreviations:** ACAN, aggrecan; COL1, collagen type I alpha 2 chain; COL2, collagen type II alpha 1 chain; FAM, carboxyfluorescein; fw, forward; IL1, interleukin 1 beta; IL6, interleukin 6; MMP1, matrix metallopeptidase 1; MMP13, matrix metallopeptidase 13; rev, reverse; TAMRA, tetramethylrhodamine.

### 2.5 Biochemical Analyses of Conditioned Medium

The sulfated glycosaminoglycan (GAG) content in the conditioned medium was determined using the dimethylmethylene blue (DMMB) method [20]. Levels of nitric oxide (NO) released in the conditioned medium of IVDs were determined through the concentration of its stable oxidation product, nitrite (NO_2_−). Nitrite concentrations were assessed spectrofluorometrically using the Griess Reagent System according to the manufacturer's instructions (Griess Reagent Kit, Promega, Madison, WI, USA).

### 2.6 Safranin O/Fast Green Staining

From a separate set of experiments, IVDs were harvested for histology staining. After 7 days of culture, the IVDs with endplates were fixed in 70% methanol, decalcified in deionized water containing 12.5% EDTA and 1.25% sodium hydroxide for 2 weeks, paraffin‐embedded, and cut sagittally into 6 μm thick sections (HM 355S, Microm, Germany). Sections were stained with 0.1% Safranin O and 0.02% Fast Green to reveal proteoglycan and collagen deposition, respectively, and counterstained with Weigert's Hematoxylin to reveal cell distribution.

### 2.7 LDH Staining

After 7 days of culture, whole IVDs were snap‐frozen in a cryo‐embedding compound (Tissue‐Tek, O.C.T., Sismex, Horgen, Switzerland) after removal of the intact endplate from one side. Transverse sections (10 μm) were cut with a microtome (HM 500 OMV, Microm, Germany). Cell viability was determined using lactate dehydrogenase (LDH) and ethidium homodimer‐1 (EthH) staining as described previously [21]. The LDH assay detects LDH activity in viable cells by conversion of nitrotetrazolium blue chloride (NBT) into a nonsoluble blue precipitate (formazan) on histological sections. Staining was performed with ethidium homodimer (1 μg/mL) and lactate dehydrogenase in 40% polypep solution (Sigma‐Aldrich, Buchs, Switzerland). Images were acquired with Zeiss LSM 510 in fluorescence and transmitted light. Blue or blue/red staining indicates living cells, while red‐only staining indicates dead cells. Axiovision software (Zeiss, Germany) was used to select regions (533 × 533 μm) of interest. Five regions per disc (the outer and inner AF from concave and convex sides, and the core of NP) were selected for imaging. The living and dead cells in each region were labeled with the software tool and counted to assess the percentage of viable cells.

### 2.8 Statistical Analysis

SPSS 21.0 software was used for statistical analysis. One‐sample Kolmogorov–Smirnov test was used to define whether the data were normally distributed (normal distribution at *p* > 0.1). For data that were normally distributed, one‐way ANOVA with Tukey post hoc test was used to determine differences among three or more groups; an unpaired *T* test was used to determine differences between two groups. For data that were not normally distributed, Kruskal–Wallis test was used to determine differences among three or more groups; Mann–Whitney U test was used to determine differences between two groups. A *p*‐value < 0.05 was considered statistically significant; *p*‐value < 0.1 was considered to indicate a trend. The numbers of biological replicates (*N*) and technical replicates (*n*) for each assay are shown in the figure legends.

## 3 Results

### 3.1 Disc Height Change

**FIGURE 2:**
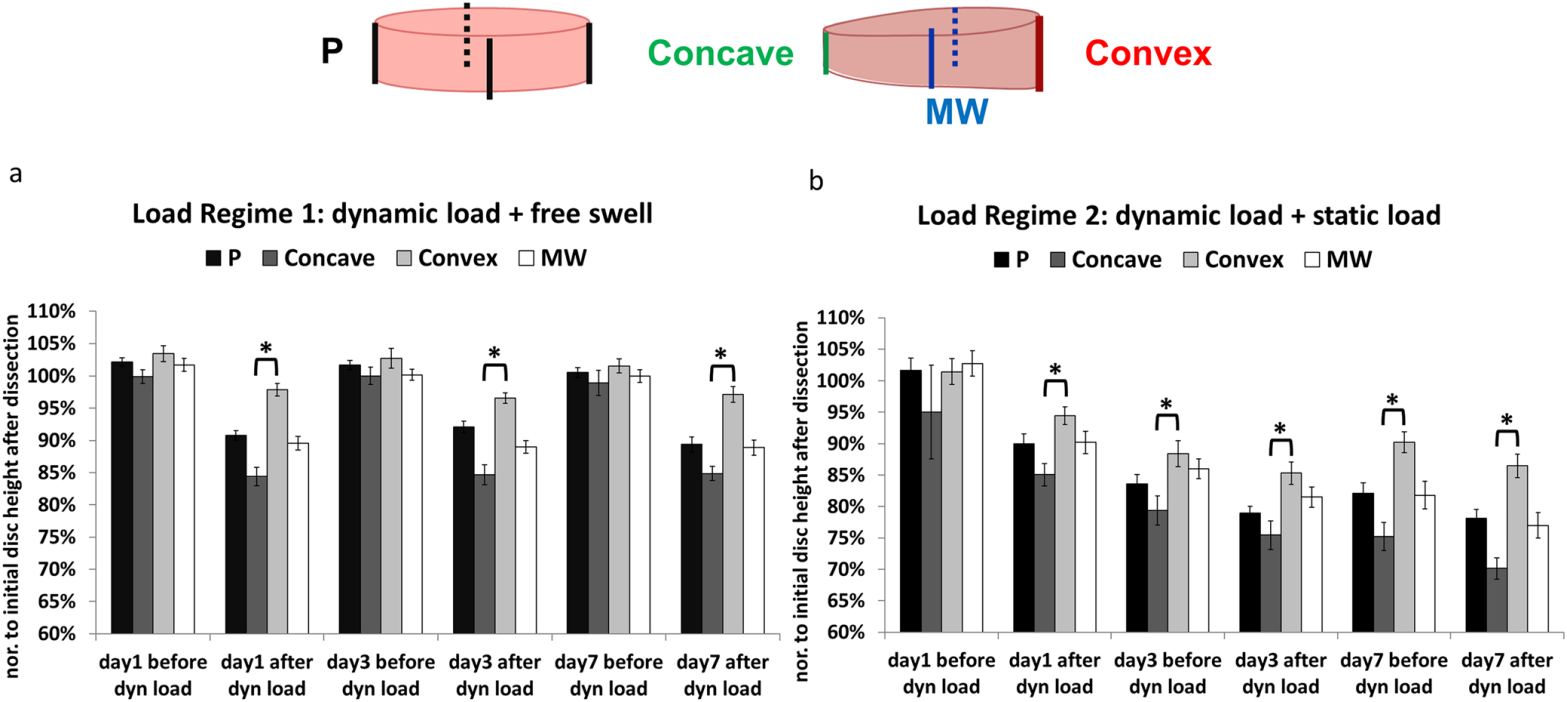
Disc height change of IVDs under different loading regimes, *N* = 3, *n* = 9–13, *p < 0.05, mean ± SEM. MW, middle of wedge IVD; P, parallel.

Under dynamic load + free swelling load regime, for both Parallel and Wedge groups, the disc height decreased after dynamic loading and could recover to the initial disc height after free swelling throughout the entire culture period of 7 days. This means the wedged IVD shape in the wedge group was only maintained during the dynamic loading period. The disc height in the parallel group decreased by 10% after dynamic loading. In the wedge group, the disc height at the concave side decreased by 15% after dynamic loading and decreased by about 5% at the convex side (Figure 2a). Under dynamic load + static load regime, the disc height of the parallel group decreased by 20% after 7 days of loading. The concave side of the wedged disc even decreased by 30%, while the convex side of the wedged disc decreased by 15%. The wedged shape was maintained during the entire culture period (Figure 2b).

### 3.2 Gene Expression

**FIGURE 3:**
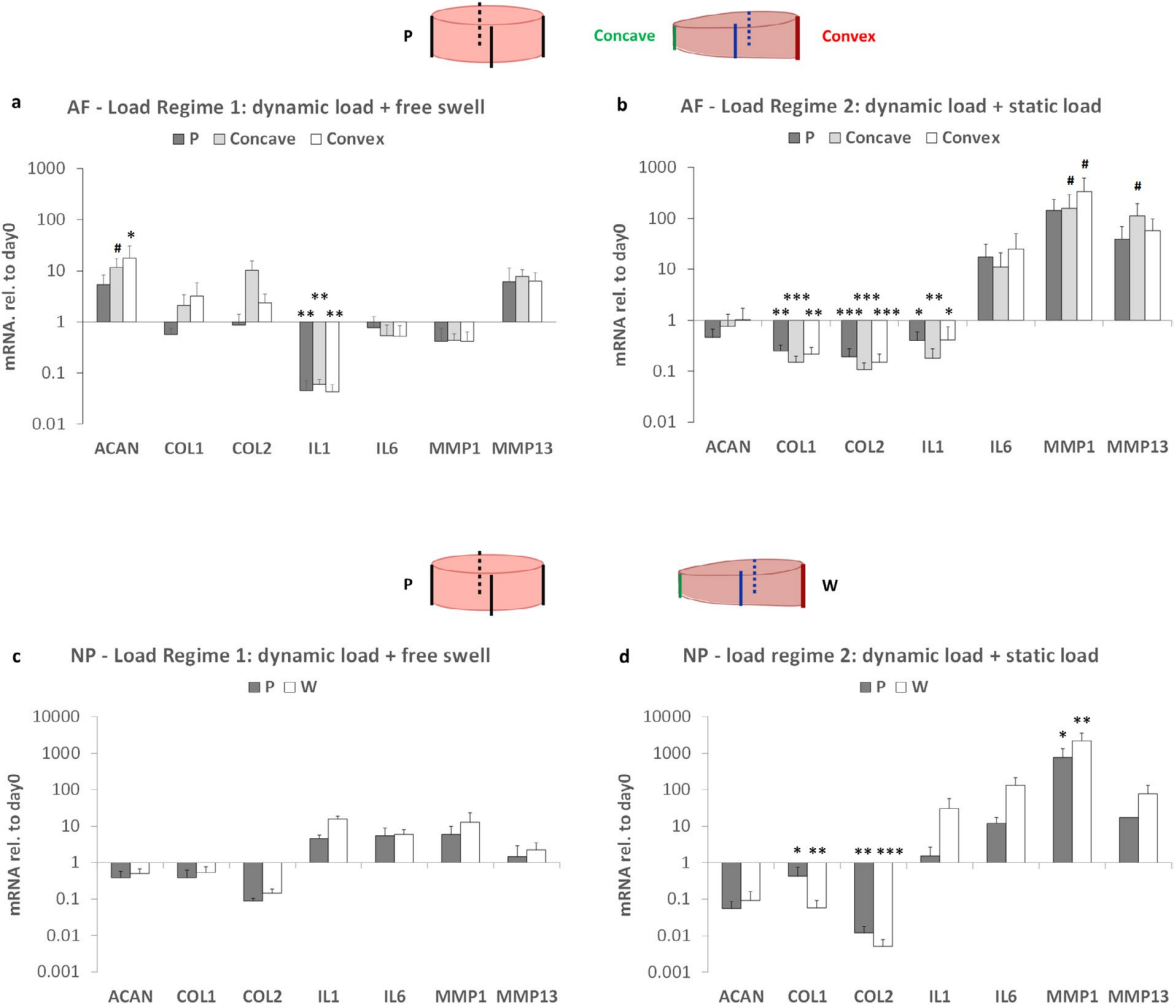
Relative mRNA expression of AF (a, b) and NP (c, d) tissue of parallel or wedge‐loaded IVDs under dynamic load + free swelling (a, c, *N* = 3, *n* = 6) or dynamic load + static load condition (b, d, *N* = 3, *n* = 11) after 7 days of culture. Data were normalized to the gene expression level of disc tissue from respective bovine tail before starting organ culture on day 0. #*p* < 0.1, *p < 0.05, **p < 0.01, ***p < 0.001 compared with day 0, mean + SD.

The AF tissues from the parallel group, and the concave and convex sides of the two wedge groups were collected for gene expression measurement of several anabolic markers: ACAN, COL1, COL2; catabolic markers: MMP1 and MMP13, and inflammatory markers: IL1 and IL6 (Figure 3). Comparing the two load regimes, under the dynamic load + static load regime, the anabolic genes showed significantly lower expression, and the inflammation and catabolic genes showed higher expression, which indicates that the dynamic load + static load regime mimics a more degenerative condition for the IVD compared with the dynamic load + free swelling regime. The differences between the Parallel and wedge groups are also influenced by loading regimes: under the dynamic load + free swelling regime, the ACAN gene expression showed an increased level in the concave and convex sides of the wedge group. Under the dynamic load + static load regime, the MMP1 gene expression showed a trend of increase in the concave and convex sides of the wedge group; the MMP13 gene expression showed a trend of increase in the concave side of the wedge group.

The NP tissues from the Parallel and Wedge groups were also collected for gene expression measurement of the same set of genes. Comparing the 2 load regimes, the NP tissue showed the same trend as the AF tissue: anabolic genes were down‐regulated, inflammation and catabolic genes were upregulated under dynamic load + static load regime. No difference was observed comparing Parallel and Wedge groups under either load regime.

### 3.3 GAG and NO Content in Conditioned Medium

**FIGURE 4:**
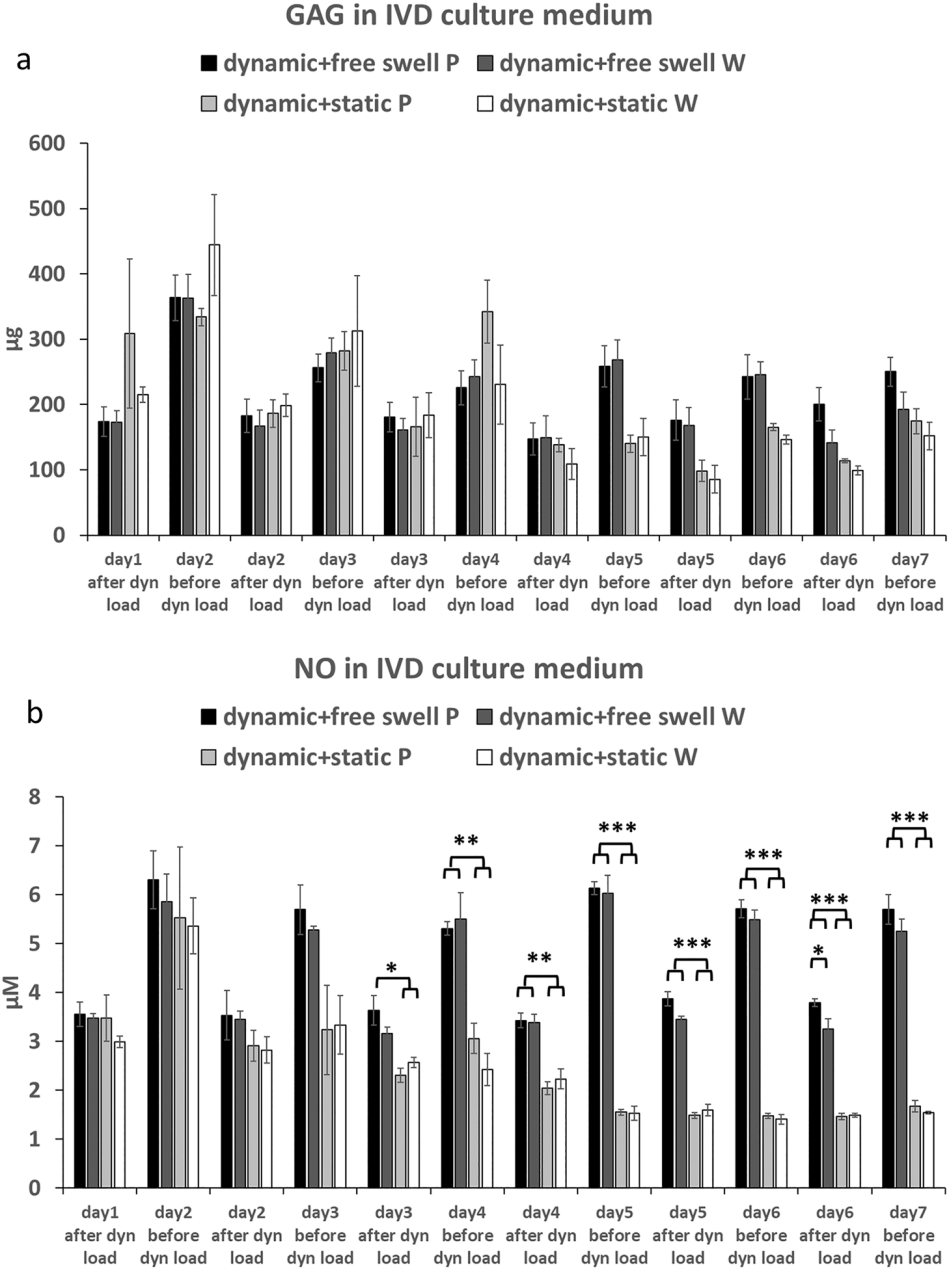
GAG (a) and NO (b) release in IVD culture medium during dynamic loading (after dyn load) and free swelling or static loading culture (before dyn load), mean ± SEM, *N* = 3, *n* = 9, *p < 0.05, **p < 0.01, ***p < 0.001.

GAG content in conditioned medium did not show any significant difference among the 4 groups at all time points (Figure 4a). Starting from day 4, the NO concentration in conditioned medium showed a higher value under dynamic load + free swelling regime compared with dynamic load + static load regime (Figure 4b).

### 3.4 Safranin O/Fast Green Staining

**FIGURE 5:**
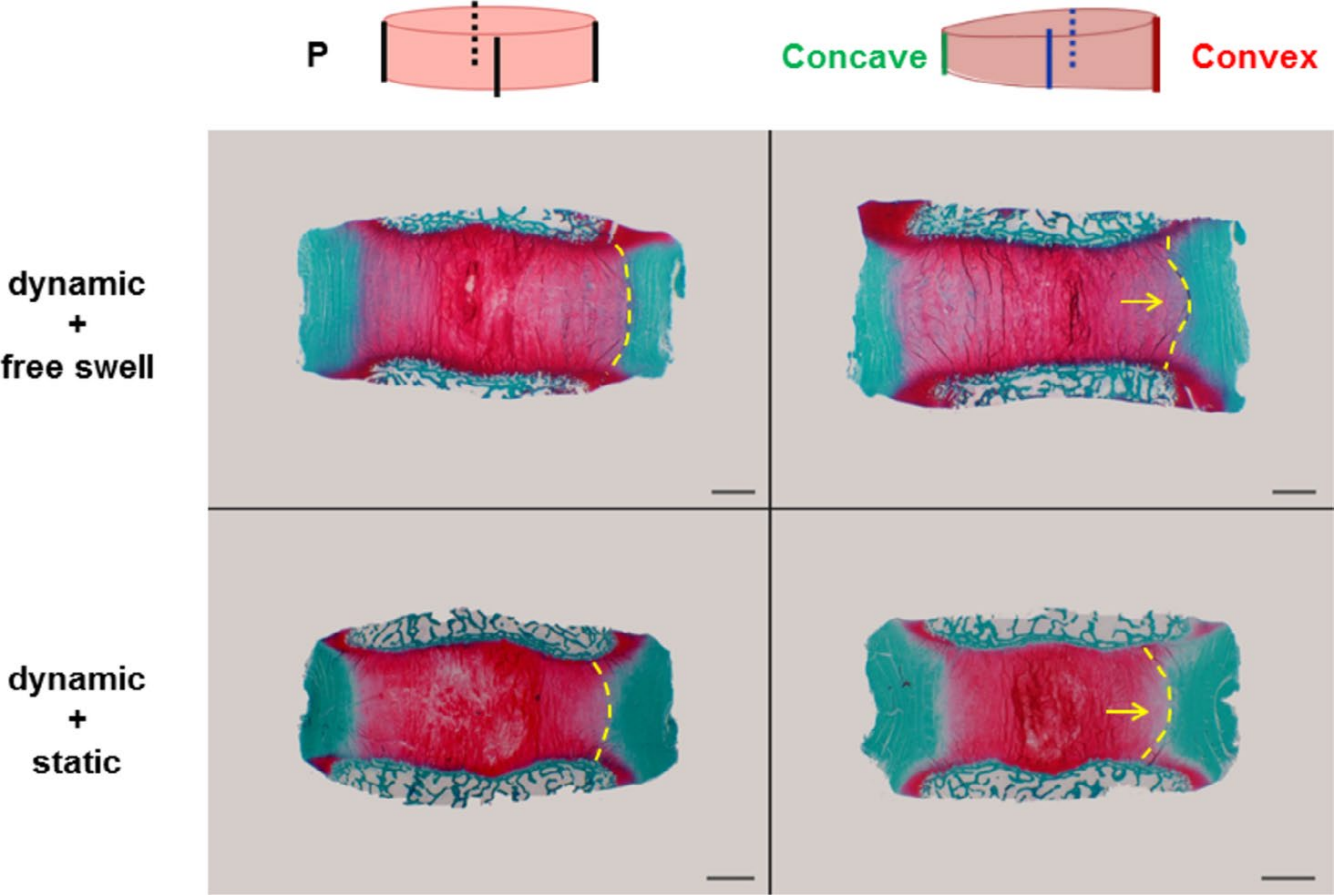
Representative Safranin O/Fast Green staining images of sagittal sections of IVDs. Bovine coccygeal IVD organ explant cultured with different loading regimes for 7 days and 6 μm sections from EDTA‐decalcified and paraffin‐embedded tissue. Scale bar: 2 mm.

**FIGURE 6:**
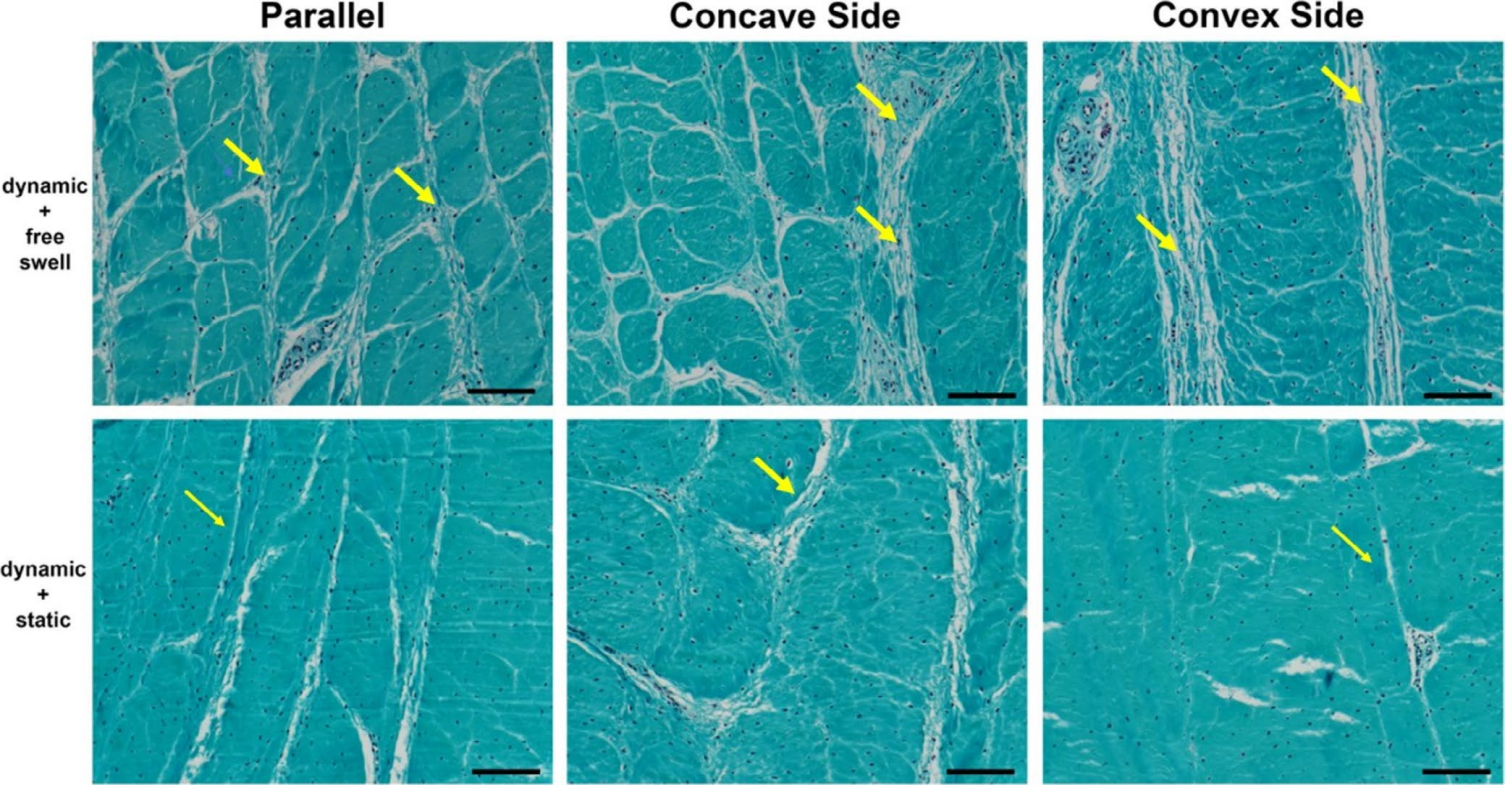
Representative Safranin O/Fast Green staining images of annulus fibrosus tissue in parallel loaded, Concave and Convex side of wedge‐loaded IVD, after 7 days of culture under dynamic load + free swell or dynamic load + static load culture condition, decalcified with EDTA and cut at 6 μm sections. The yellow arrows indicate the intralaminar spacing within the AF tissue. Scale bar: 100 μm.

Images of Safranin O/Fast Green stained sections of IVDs cultured for 7 days are shown in Figures 5 and 6. The staining intensity of proteoglycan (red) in NP tissue and collagen (green) in AF tissue was comparable among the 4 groups. The NP tissue in the wedge group, in both loading regime groups, showed a trend of protrusion toward the convex side (Figure 5). The IVDs cultured under dynamic load + free swelling condition showed larger intralaminar spacing within the AF tissue, compared with the IVDs cultured under dynamic load + static load condition (Figure 6).

### 3.5 Cell Viability

**FIGURE 7:**
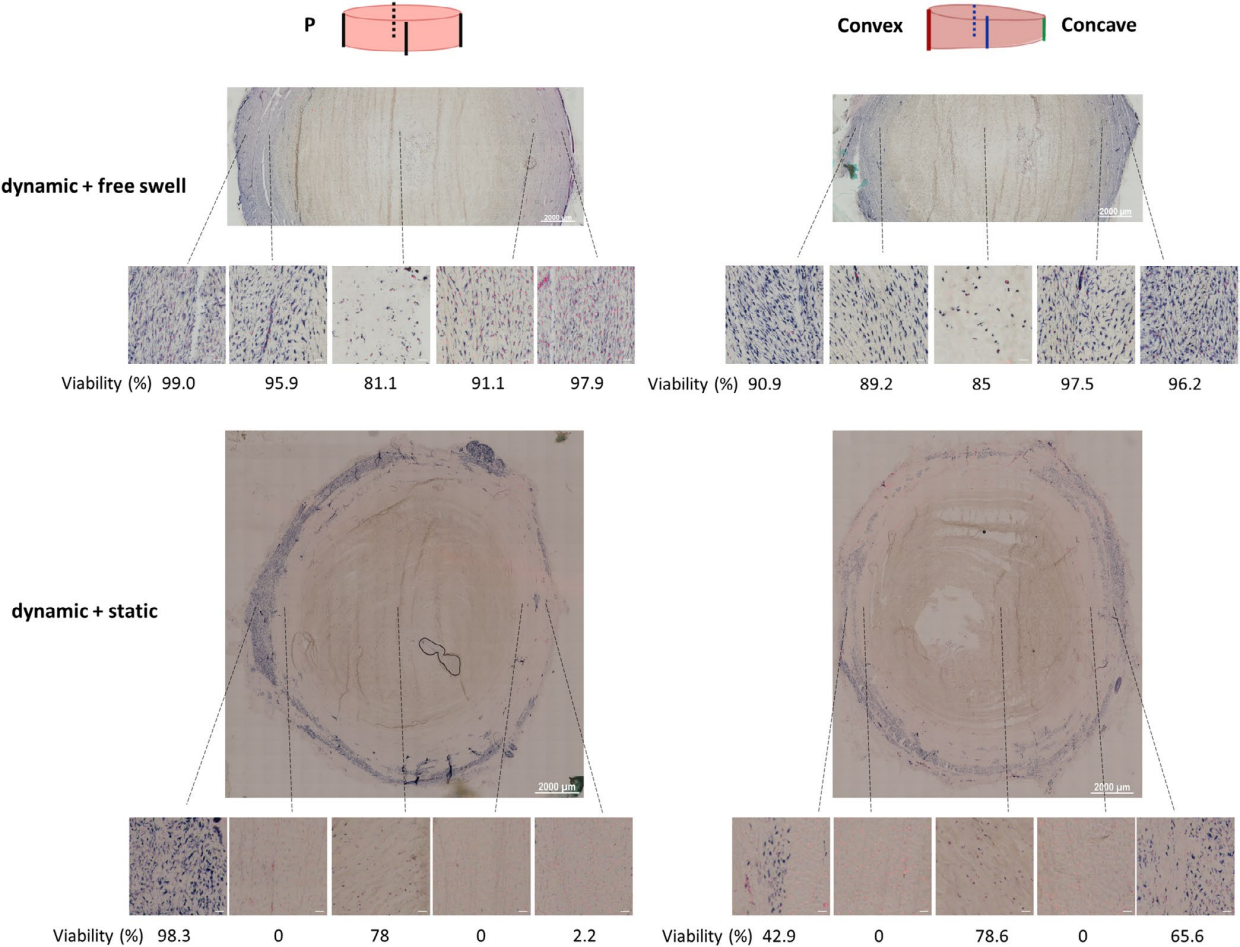
Cell viability assessed using LDH‐ethidium homodimer staining (blue and blue/red = living cell, red only = dead cell) on transverse IVD cryo‐sections with 10 μm thickness. Representative images of the outer AF, inner AF and NP are reported. Scale bar: Overview image—2000 μm, regional image—50 μm.

In both Parallel and Wedge groups under dynamic load + free swelling condition, the cell viability remained high (above 80%) after 7 days of culture in the outer AF, inner AF, and NP regions. The cell viability in the NP (~80%–90%) was slightly lower than in the AF (~90%–100%) (Figure 7 upper row). Under dynamic load + static load condition, the cell viability in the inner AF region dropped dramatically to 0%, independent of the Parallel or Wedge loading pattern. The cell viability in the outer AF decreased in random regions under dynamic load + static load condition, independent of the Parallel or Wedge loading, and also independent of the Concave or Convex side. The cell viability in the NP region was maintained above 75% under dynamic load + static load condition (Figure 7 lower row).

## 4 Discussion

With the aim of finding relevant structural, cellular, and biochemical changes in asymmetrically loaded discs, bovine coccygeal IVDs were compressed with parallel dynamic load or wedge dynamic load followed by free swelling or static load within a custom‐made chamber. Structural and biological changes were assessed with disc height, gene and protein expression, GAG, and NO release. Histology provided insight into localized cell viability and tissue morphology changes. Results were compared with other relevant loading models from the literature and the pathology of spinal deformity.

The current study validated a large animal whole organ culture model of asymmetric loading with a custom‐made chamber and wedge spacers. IVDs loaded within the chamber remained at the starting position without sliding or rotation. Furthermore, the wedged shape was maintained under dynamic or static loading, as indicated by the disc height measurement at the concave and convex sides. Previous studies investigating the effect of asymmetric loading on IVDs were performed in a static setting using either rat tail IVD [22] or bovine caudal IVD [14]. In contrast, the current study established a novel large animal IVD organ dynamic asymmetric loading model.

The loading regime is related to phenotypic changes of IVD cells. In this study, lower anabolic gene (ACAN, COL1, COL2) and higher catabolic gene (MMP) expression were detected in IVDs loaded with diurnal dynamic loading followed by static loading. On the other hand, higher anabolic gene expression was detected in IVDs subjected to diurnal dynamic loading and free swelling. Increased expression of MMPs (MMP‐1, ‐2, ‐3, ‐7, ‐8, ‐10, ‐12 and ‐13) has been confirmed in degenerative discs [23, 24, 25, 26]. Sustained static compression loading in rat tail has shown to upregulate catabolic gene expression of MMPs in the IVD tissue [25]. Our results are consistent with the literature, which further proved that sustained static loading induces degeneration cascades in the IVD.

Asymmetric loading of IVDs is very common. Normal spine has three curves on the sagittal plane: cervical lordosis, thoracic kyphosis, and lumbar lordosis. Cervical lordosis is usually represented by Cobb's angle formed by the lower endplate of C2 and C7. These curves are formed by the subtle wedged shape of every IVD. Although asymmetric load presents at all spine segments, thoracic IVD showed rare degeneration. It is because the thoracic spine serves as a relatively rigid part of the chest cage, while the cervical and lumbar spine has large movements. Asymmetric load may also be static, such as in patients with scoliosis. In adult degenerative scoliosis, which implies a very long period of asymmetric loading, the concave side of the asymmetrically loaded IVD usually shows more severe degenerative signs, such as large osteophytes or auto‐fusion, than the convex side. The physical mechanism of such differences is still undetermined. Vasiliadis et al. [18] reported that the expression of MMP12 increased in the IVD after the application of asymmetric loading in a rat tail and correlated with the degree of the deformity and the side of the wedged disc. Moreover, it has been shown that MMP13 was highly upregulated in a rat‐induced scoliosis model [27]. However, our study found that degenerative changes were due to constant load but not the load pattern (parallel or wedge), at least in a procedure of 7 days.

Previous studies have demonstrated a correlation between spinal loading and cell death. After 28 days of static compression, significantly increased numbers of dead cells were observed in the AF of rabbits, compared to those treated with dynamic compression or distraction [28]. Lotz and Chin [29] further developed a formula to estimate the quantitative relationship of cell viability with loading duration and magnitude, which showed that higher pressure and longer duration are related to more dead cells. The present study reinforces the previously established concept that non‐physiological spinal loading induces cell death. Dynamic load + free swelling maintained the cell viability above 80% in the NP, inner, and outer AF regions. Dynamic + static load dramatically reduced the cell viability in certain regions of the outer AF tissue and the whole region of the inner AF tissue. Our recent research also found that the inner AF had a lower cell viability compared with outer AF and NP under cyclic compressive loading in the uniaxial bioreactor, which indicates that the cells in the inner AF region may be more sensitive to detrimental loading conditions [9].

Other research also found that different areas of IVDs reacted differently under load, but there was no consensus on which area was more sensitive. Our research found that more cell death happened in the inner AF region, under 1 h dynamic compression of 0.02–0.4 MPa followed by free swelling or static compression of 0.2 MPa in the present study, or 2 h dynamic compressive loading of 0.02–0.2 MPa, 0.2 Hz followed by overnight free swelling [9]. Paul et al. [30] cultured goat lumbar IVDs under high dynamic overloading (0.4–0.8 MPa, 1 Hz, 16 h) or high static overloading (0.6 MPa, 16 h) conditions, both followed by 8 h of 0.1 MPa static load for 14 days, and assessed the response in the NP, inner AF, as well as anterior, lateral, and posterior outer AF regions. High dynamic overloading caused cell death in all IVD regions, whereas high static overloading particularly affected cell viability in the posterior region of the outer annulus. Another study using bovine caudal IVDs found that both the NP and the AF tissues showed a correlation between the intensity of the loading regimes and the extent of cell death, so that the loading regimes with increased amplitude also led to an increase in cell death [31]. The static loading was 0.1 MPa for 7 days, while low stress was set as 0.1 MPa for 16 h + 0.1–0.2 MPa with twist for 8 h, intermediate stress up to 0.4 MPa, and high stress up to 0.6 MPa. No living cells were detected within the NP when the high‐stress regime was applied. These observations indicate that different loading, including magnitude, duration, pattern, and regime, implies different influences on IVD regions, and detailed research is needed to find the threshold for inner AF, outer AF, and NP in response to different types of load.

It is possible that static loading induced cell death by provoking catabolic and inflammatory responses with mRNA upregulation of MMPs, IL1, and IL6 [32, 33], which was also verified in our study. In the current study, cell viability comparison of the concave side (65.6%) and convex side (42.9%) of IVDs revealed slightly more dead cells on the convex side. A previous study with static asymmetric loading of 7 days found more dead cells on the concave side [14]. Additionally, in a study where scoliosis was induced in immature goats by spinal tethering and clamping for 6 months, cell density was significantly decreased toward the concave side [34]. Such contradiction emphasizes the different influences of loading modes, like continuous static load, intermittent static load, dynamic load, or their combination.

IVDs treated with dynamic and static load, in both Parallel and Wedge dynamic load + static load groups, established a lower swelling and medium absorption rate. The lower GAG and NO release into the conditioned medium, compared with Parallel and Wedge dynamic load + free swelling groups, may be due to this impaired molecule exchange between the disc tissue and medium. Together with the significantly lower live cell numbers and irretrievable disc height, in both Parallel and Wedge dynamic load + static load groups, while there was no significant difference between these two groups, we may draw the conclusion that continuous load has a stronger influence on IVD biological changes than symmetric or asymmetric load pattern, at least in a relatively short period of 7 days.

Shortcomings of the present study mainly resist in the difference between human lifestyle and experiment set‐up. Homo erectus bears much bigger loads in their spines than all other animals. Wilke et al. [35] used an invasive transducer and found that human IVDs hold a pressure of 0.1–0.24 MPa during the night in one volunteer, not a load‐free condition. Both clinical data and experimental techniques need to be improved to further study the mechanical effect on the metabolism of human IVDs. Another limitation is that the effect of Cobb's angle is not determined in this study, as this study is a single “segment” analysis of the IVD organ.

Our findings suggest that diurnal dynamic loading and free swelling recovery could maintain the gene expression of organ‐cultured discs at their physiological level and preserve cell viability. Diurnal dynamic loading followed by static loading mimicked a degenerative condition, as indicated by lower anabolic and higher catabolic gene expression and lower cell viability. These results indicate that physical treatment to reduce the stress on the IVD, even temporarily, may help to prevent the acceleration of deformity or degeneration. Asymmetric dynamic and static loading mimics the deformation changes of the IVD from scoliosis patients. Further studies are required to elucidate the impact of the wedged dynamic loading duration and the status of the disc on the severity of degeneration. The unique whole organ IVD model under imbalanced loading may be used to identify molecular biomarkers and test potential reparative strategies.

## Author Contributions

Y.Z. and Z.L. performed the laboratory work and data analysis. R.G.R., M.A., Z.Z., Z.L., and S.G. contributed to the study design and critically revised the manuscript. Y.Z., Z.L., and J.X. contributed to manuscript drafting. All authors read and approved the final manuscript.

## Conflicts of Interest

The authors declare no conflicts of interest.
